# Core Muscle Activation With Foam Rolling and Static Planks

**DOI:** 10.3389/fphys.2022.852094

**Published:** 2022-03-08

**Authors:** Ali Zahiri, Shahab Alizadeh, Abdolhamid Daneshjoo, Nick Pike, Andreas Konrad, David G. Behm

**Affiliations:** ^1^School of Human Kinetics and Recreation, Memorial University of Newfoundland, St. John’s, NL, Canada; ^2^Department of Sport Injuries, Physical Education and Sport Sciences Faculty, Shahid Bahonar University of Kerman, Kerman, Iran; ^3^Institute of Human Movement Science, Sport and Health, University of Graz, Graz, Austria

**Keywords:** trunk, abdominals, back, electromyography, erector spinae, external obliques

## Abstract

The objective of this study was to compare the activation of the core (trunk) musculature during quadriceps and hamstrings foam rolling (FR) vs. prone and supine/reverse static planks to determine if FR is a viable means of training the core musculature. Using a randomized allocation, nine recreationally trained, young adults (18–26 years) performed two sets each of quadriceps and hamstrings FR as well as supine/reverse and prone static planks for 30-s each with 1-min rest between sets and 5-min rest between exercises. Electromyographic (EMG) activity of the lower abdominals (LA), external obliques (EO), lumbosacral erector spinae (LSES), upper lumbar erector spinae (ULES) muscle groups were normalized to a maximum voluntary contraction and analyzed. Quadriceps FR exhibited a very large magnitude greater LA activity compared to reverse plank (*p* = 0.033, *d* = 4.42) and hamstrings FR (*p* = 0.020, *d* = 3.49), respectively. The prone plank demonstrated very large magnitude higher EO EMG activity compared to reverse plank (*p* = 0.001, *d* = 9.17), hamstrings FR (*p* = 0.002, *d* = 8.14), and quadriceps FR (*p* = 0.011, *d* = 5.97). Reverse plank (*p* = 0.003, *d* = 12.06), and quadriceps FR (*p* = 0.002, *d* = 7.84) induced greater ULES activity compared to the prone plank and hamstrings FR, respectively. Reverse plank also exhibited very large magnitude higher LSES activity compared to the prone plank (p < 0.001, *d* = 7.68), hamstrings FR (*p* = 0.002, *d* = 4.11), and quadriceps FR (*p* = 0.005, *d* = 2.34), respectively. In conclusion, whereas reverse plank was the most effective activator of dorsal core muscles, quadriceps FR may also be a time efficient alternative exercise to activate back (ventral core) muscles. The prone plank is effective for ventral core muscles activation.

## Introduction

Training the trunk or core musculature, has received considerable emphasis in the scientific and professional literature as well as the sports training, rehabilitation, and injury prevention fields (Anderson and Behm, 2005a; Behm and Anderson, 2006; Behm et al., 2010a,b, 2011; Behm and Colado, 2012). The anatomical core refers to the axial skeleton (including the pelvic and shoulder girdles) and the associated soft tissue (e.g., ligaments, tendons, and muscles) (Behm et al., 2010a), that act to generate (concentric action) or resist motion (eccentric and isometric actions) and maintain the stability of the spine and pelvis when performing both simple and complex movements (Behm and Anderson, 2006; Kibler et al., 2006; Behm and Colado Sanchez, 2013). The appropriate intensity and sequential activation patterns of core musculature also play an essential role in the stability of the vertebral column and the effective transfer of torques and angular momentum through the kinetic chain (Behm et al., 2010a; Behm and Colado Sanchez, 2013), as well as reducing musculoskeletal injury risk (Kibler et al., 2006).

To ensure that athletic movements or activities of daily living are performed without the risk of injury or unnecessary muscle soreness, the core muscles need to be appropriately conditioned (Willardson, 2007a,b; Behm et al., 2010a,^2011^, ^2015^). For example, low back pain is one of the most common musculoskeletal health problems internationally (Steffens et al., 2016) and is associated with the deconditioning of the lumbar extensors (Behennah et al., 2018). Despite the bulk of evidence in support of training the trunk musculature (Willardson, 2007a,b; Behm et al., 2010a,^2011^; Sipaviciene and Kliziene, 2020), no consensus exists regarding the optimal exercises for training these muscles (Willardson, 2007a,b; Behm et al., 2010a,b, 2011). However, numerous exercises and training techniques for the trunk muscles do exist. Various prone and supine callisthenics-type exercises (e.g., static plank, back extensions, and sit-ups) can activate the trunk muscles (Parfrey et al., 2008, 2014; van den Tillaar and Saeterbakken, 2018).

Another exercise and rehabilitative potential training method for the core musculature could be foam rolling (Junker and Stoggl, 2015; Behm and Wilke, 2019; Behm et al., 2020; Nakamura et al., 2021) especially when it is applied on the lower leg muscles. Foam rolling is a form of rolling self-massage in which body weight is applied on a foam roller to roll and compress the targeted musculature (MacDonald et al., 2013; Pearcey et al., 2015; Wiewelhove et al., 2019; Wilke et al., 2020). Proposed acute benefits of foam rolling include increased joint range of motion (Behm and Wilke, 2019; Behm et al., 2020; Wilke et al., 2020; Nakamura et al., 2021), and in some studies improved performance (Halperin et al., 2014; Skinner et al., 2020; Reiner et al., 2021).

However, a gap within the foam rolling literature is how foam rolling affects the activation of core musculature, foam rolling in many cases, necessitates supporting the body weight with the upper body, similar to planking exercises (e.g., foam rolling the legs). Planking exercises involve isometrically holding the body in a prone or supine position to strengthen and improve the endurance of the core muscles (van den Tillaar and Saeterbakken, 2018). However, unlike planking exercises, foam rolling involves moving one’s partial bodyweight dynamically, rather than isometrically, on a foam roller to roll and compress the targeted musculature. projecting the body’s center of mass beyond a foam rolling area of support can induce periodic instability [metastability: (Kibele et al., 2015)], which may increase core muscle activation (Behm et al., 2002a,2010a; Anderson and Behm, 2005b). if foam rolling is an effective method of activating the trunk musculature, additional trunk-specific exercises such as planks, sit-ups, and back extensions may not be necessary and hence, this would decrease the training time. A lack of time has been cited as a significant barrier to regular exercise (Canadian Society for Exercise Physiology, 2003). Identifying exercises that involve and integrate more muscle groups could decrease exercise time (Iversen et al., 2021), encourage more consistent activity and benefit the health of the general population.

Therefore, this study’s objective was to compare core (trunk) muscle activation during foam rolling (quadriceps and hamstrings) to that of a static plank (prone and reverse) to determine if foam rolling is a viable means of training the core musculature. Based on research examining core activation with dynamic and rhythmic resistance training under stable and metastable conditions (Behm et al., 2002a,2010b,^2015^; Behm and Anderson, 2006; Behm and Colado, 2012), it was hypothesized that foam rolling would elicit higher levels of core (trunk) activation (EMG) than a static plank exercise.

## Materials and Methods

### Experimental Design

The experiment used a within-subjects, repeated measures, randomized, design. The participants were monitored for the root mean square (RMS) amplitude of the EMG activity of the lower abdominals, lumbosacral erector spinae (LSES), upper lumbar erector spinae (ULES), and external oblique muscle groups for 30-s while foam rolling the (1) quadriceps and (2) hamstrings of the dominant leg, as well as maintaining a static plank in a (3) prone or (4) supine position (commonly referred to as the reverse plank). A 5-min rest period was allocated between exercises. EMG activity was normalized to a maximum voluntary isometric contraction (MVIC) for each muscle group.

### Participants

Participants were recruited using convenience sampling by word of mouth and posters. An “a priori” statistical power analysis (G*Power: University of Dusseldorf) was performed using EMG data from two studies (Anderson and Behm, 2005b; Behm et al., 2005) to determine the appropriate sample size for this experiment. Based on the analysis, a sample size of 4–10 participants was needed to achieve an alpha level of 0.05, effect size of 0.5, and a statistical power of 0.8. Hence, nine male participants volunteered (age: 29.3 ± 5.1 yrs., height: 174.7 ± 4.9 cm, weight: 73.6 ± 8.3 kg). Inclusion criteria for participants included recreationally trained (participate in physical activity ≥ 3 times⋅wk^–1^) adults between the ages of 18 and 40 who could complete a prone and supine/reverse static plank and actively engage in foam rolling of the quadriceps and hamstrings for at least 30-s. Exclusion criteria for participants included any existing musculoskeletal injuries or back pain. Before any testing session, participants were asked to avoid vigorous physical activity and refrain from alcohol consumption for at least 24 h. Participants were also asked to avoid tobacco and caffeine containing products for at least 4 h prior to each session. All participants recruited for this study were verbally informed of all procedures. If willing to participate, they read and signed a written consent form before participation. Participants were also required to complete the Physical Activity Readiness Questionnaire to rule out potential health issues. Approval from the institution’s Interdisciplinary Committee on Ethics in Human Research was obtained (2021-0626-HK) in accordance with the Declaration of Helsinki.

### Experimental Sessions

The first session was an orientation session which included foam rolling and MVIC familiarization. When participants arrived, they signed a written consent form and completed a health status questionnaire. Participants were then familiarized with the EMG normalization protocols (MVIC for each monitored muscle group), location of EMG electrodes (lower abdominals, LSES, ULES, and external obliques) as well as the foam rolling and plank exercises. All the participants were provided with verbal instructions and received an illustration about procedure and tasks.

After a similar familiarization session in the second session, participants were prepared for electrode placement. Normalization protocols were preceded by a warm-up on a Monark cycle ergometer (Monark Exercise AB, Vansbro, Sweden) at an intensity of 1 kilopond at 70 revolutions per minute (rpm) for 5 min. Following the EMG MVIC normalization procedures, participants completed each experimental foam rolling and plank protocol in a random order. The second experimental session included the completion of 30-s foam rolling and plank protocols with 5-min rest between exercises.

### Electromyography

Bipolar surface EMG electrodes were used to monitor lower abdominals, LSES, ULES, and external oblique muscle activity during the tasks. All electrodes were placed collar to collar (approximately 2 cm) on the dominant side of the body. Skin surfaces for electrode placement were shaved, abraded, and cleansed with alcohol to improve EMG signal conductivity. Electrode placement followed the guidelines used by Behm et al. (2006). Electrodes were placed 2 cm lateral to L5-S1 spinous processes for the LSES and 6 cm lateral to the L1-L2 spinous processes for the ULES muscles. Additional electrodes were placed superior to the inguinal ligament and 1 cm medial to the iliac crest for the lower abdominal muscles. Electrodes for the external obliques were placed 3 cm medial to a line midway between the floating ribs and the anterior superior iliac spine in an oblique positioning following the direction of the external oblique muscle fibres. A ground electrode was placed along the lateral tibial tuberosity.

Electromyography was sampled at 2000 Hz, filtered with a Blackman 61 dB band-pass filter between 10 and 500 Hz, amplified (bi-polar differential amplifier, input impedance of 2 MOhms, common mode rejection ratio of 110 dB min (50/60 Hz), gain of 1000), (Biopac Systems MEC 150 amplifier, Santa Barbara, California), and stored for further analysis. The baseline resting EMG signal was monitored at the start of each testing session to ensure it was less than 0.05 millivolts (mV). Each EMG signal was rectified and smoothed (ten samples) using the AcqKnowledge software program (Biopac Systems, Santa Barbara, California). The mean amplitude of the RMS EMG signal was calculated over the full 30-s duration of each activity with each 10-s period analyzed separately (i.e., 0–10, 10–20, and 20–30-s). The reliability of this EMG measurement technique has been demonstrated by this laboratory in previously published literature with reported intraclass correlation coefficient values ranging from 0.91 to 0.99 (Behm et al., 2001, 2002b).

Electromyography activity for the LSES, ULES, lower abdominals, and external oblique muscle groups were normalized using distinct normalization procedures. A manually resisted maximum isometric back extension in the position of a modified Biering-Sorensen test was used to determine maximum LSES and ULES EMG activation as described by Pitcher et al. (2007, 2008) and Behm et al. (2009). The normalization procedure for the external obliques also employed a modified Biering-Sorensen test albeit with the participant lying on their non-dominant side and performing a lateral flexion MVIC. An abdominal hollowing exercise was performed to determine maximum lower abdominals activation (Behm et al., 2009). In this position, subjects were instructed to contract their abdominal muscles up and back toward their spine and attempt to pull their anterior superior iliac spine together (Parfrey et al., 2008, 2014; Behm et al., 2009). Two trials for each normalization procedure were performed with 1 min rest between trials and the trial with the highest RMS EMG mean amplitude was used for normalization. If the second MVIC exhibited RMS EMG activity that was 5% or higher than the previous two contractions, a third MVIC was performed. The MVIC was held for 5-s, and the participants were verbally encouraged throughout each MVIC contraction. The EMG activity was analyzed in the middle 3-s (i.e., 1–4 s) of the 5-s exercise (Behm et al., 2009).

### Independent Variables

#### Foam Rolling Technique

**FIGURE 1:**
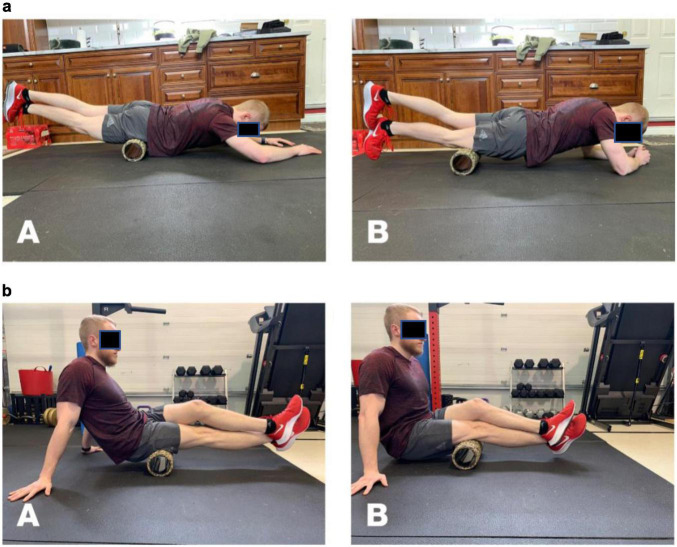
**(a)** Quadriceps foam rolling protocol. Starting position at proximal quadriceps **(A)** and end position at patella **(B)**. **(b)** Hamstrings foam rolling protocol. Starting position at proximal hamstrings **(A)** and end position at popliteal fossa **(B)**.

An extruded foam roller covered by a ridged wrap (Theraband Pro foam roller: Performance Health, Akron Ohio, United States) was used in this study for all foam rolling procedures. The device measured 33 cm in length and 14 cm in diameter and weighed 0.65 kg. Using a random allocation, participants rolled the quadriceps (Figure 1a) and hamstrings (Figure 1b) of their dominant leg. For the quadriceps roll, participants started in a modified plank position with the foam roller at the most proximal position of the quadriceps of the dominant leg with their non-dominant leg crossed over the dominant leg. Participants were asked to place as much of their body weight as possible onto the foam roller. They were then instructed to roll the foam roller down the quadriceps to a position just above the patella. Once the foam roller reached the patella, they were told to roll the foam roller back to the starting position. With the hamstrings rolling position, participants began in a seated upright position on the floor with the foam roller at the most proximal position of the dominant hamstrings. They then crossed their non-dominant leg over the dominant leg and placed as much of their body weight as possible on the foam roller. Participants were instructed to roll the foam roller down the hamstrings to a position just above the popliteal fossa. Once the foam roller reached the popliteal fossa, they were told to roll the foam roller back to the starting position. For both positions, participants repeated the rolling movements (2s up and 2s down) for 30-s in accordance with a metronome. Each participant completed two sets for both positions. The pace and total duration for the foam rolling positions followed the recommendations by Behm et al. (2020).

#### Prone and Supine/Reverse Static Planks

**FIGURE 2:**
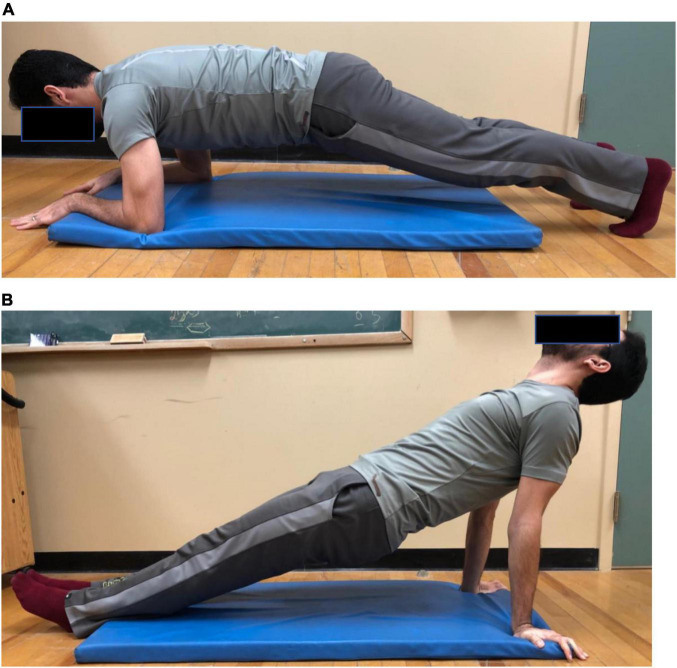
**(A)** Prone static plank position. **(B)** Supine / reverse static plank position.

Foam rolling and plank exercises commenced 5-min after the normalization procedures. With a random allocation, each participant completed the foam rolling and plank procedures with 5-min rest between exercises to ensure adequate recovery. For the prone plank, participants were instructed to assume a traditional prone plank position (van den Tillaar and Saeterbakken, 2018) on an exercise mat with their elbows flexed at 90° with only the forearms and toes in contact with the ground (Figure 2A). With the supine/reverse plank position, participants maintained an extended trunk and leg position with their extended arms placed on the floor behind and beneath their shoulders (Figure 2B). Distal support was provided by heel contact with the floor. While in these positions, participants were instructed to maintain a rigid torso, neutral head and spine, and extended leg position.

#### Statistical Analysis

Statistical analyses were calculated using SPSS software (Version 16.0, SPSS, Inc, Chicago, IL, United States). This study employed a repeated measure within-subjects cross-over design. Normality (Shapiro-Wilk) and homogeneity of variances (Levene) tests were conducted for all dependent variables. If the assumption of sphericity was violated, the Greenhouse-Geiser correction was employed. Significance was defined as *p* < 0.05. Modified Bonferroni post-hoc tests were conducted to detect significant differences. For repeated measures main effects and interactions, the effect sizes were tested using partial eta (pη^2^) squared (0.01 = small effect, 0.06 = medium effect, and 0.14 = large effect) (Pallant, 2007). For significant individual comparisons reveled with post-hoc tests, Cohen’s d effect size statistics were conducted to evaluate the magnitude of the changes following various exercise protocols to the criterion of ≥ 0.80 large; 0.50- < 0.80 medium, 0.2- < 0.50 small and < 0.2 trivial (Cohen, 1988). Data were analyzed separately for each muscle using a two-way repeated-measures analysis of variance (ANOVA) with four conditions (quadriceps and hamstrings foam rolling and prone and reverse planks) and three times (0-10-s, 10-20-s, and 20-30-s).

## Results

### Lower Abdominal Muscles

**FIGURE 3:**
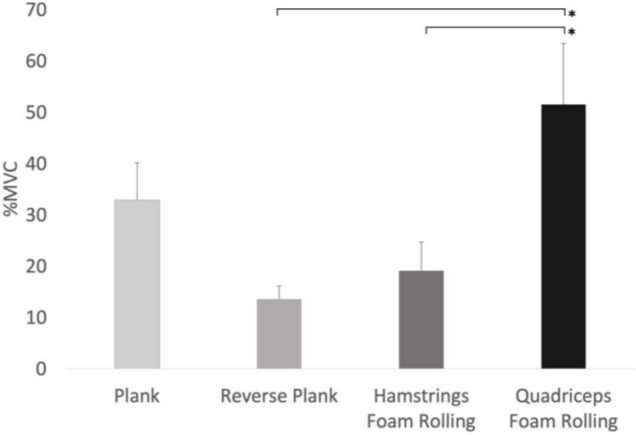
Main effect for conditions with lower abdominal muscle EMG activity. *Significant difference between the conditions (*p* < 0.05), %MVIC refers to MVICmax.

There was a main effect for condition on lower abdominal muscle activity (*F*_3,24_ = 8.644, *p* < 0.001, Pη2 = 0.519), with quadriceps foam rolling showing 279% and 170% more activity compared to reverse plank (*p* = 0.033, *d* = 4.42) and hamstrings foam rolling (*p* = 0.020, *d* = 3.49), respectively (Figure 3). Our results showed there was no main effect for time (*F*_2,16_ = 2.722, *p* = 0.096), nor was there a time × condition interaction (*F*_6,48_ = 8.644, *p* = 0.190).

### External Oblique Muscle

**FIGURE 4:**
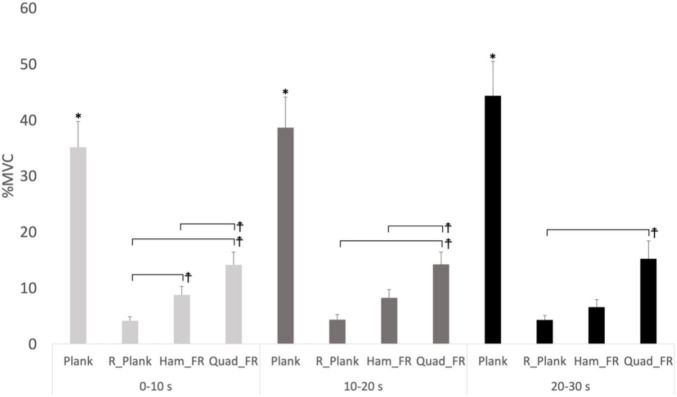
Time*Condition interaction with external oblique muscle EMG activity. *Significance difference between plank with reverse plank, hamstrings foam rolling and quadriceps foam rolling (*p* < 0.05). *Significant difference between the conditions (*p* < 0.05), %MVIC refers to MVICmax. R_Plank, reverse plank; Ham_FR, hamstrings foam rolling; Quads_FR, quadriceps foam rolling groups. The symbol “‡” represents a significant difference between the two conditions (exercises) illustrated by the horizontal lines.

There was a main effect for condition with external oblique muscle activity (*F*_3,24_ = 33.007, *p* < 0.001, Pη2 = 0.805), with the prone plank showing 823%, 403% and 171% higher EMG activity compared to reverse plank (*p* = 0.001, *d* = 9.17), hamstrings (*p* = 0.002, *d* = 8.14), and quadriceps foam rolling (*p* = 0.011, *d* = 5.97), respectively. Moreover, external oblique muscle activity during hamstrings (*p* = 0.001, *d* = 3.68, 83%), and quadriceps foam rolling (*p* = 0.003, *d* = 5.66, 240%) was higher compared to reverse plank, respectively. External obliques EMG activity with quadriceps foam rolling was greater than hamstrings foam rolling (*p* = 0.014, *d* = 3.51, 86%). A main effect for time (*F*_2,16_ = 10.080, *p* = 0.002, Pη2 = 0.558) revealed an increase in external oblique EMG activity between 0–10s and 20–30s (*p* = 0.008, *d* = 0.65). Significant time × condition interaction effects (*F*_6,48_ = 6.516, *p* = 0.004, Pη2 = 0.449) generally demonstrated that the prone plank demonstrated the greatest activity over the three time periods (0–10s, 10–20s, and 20–30s), whereas the reverse plank exhibited the least EMG activity (Figure 4).

### Upper Lumbar Erector Spinae Muscles

**FIGURE 5:**
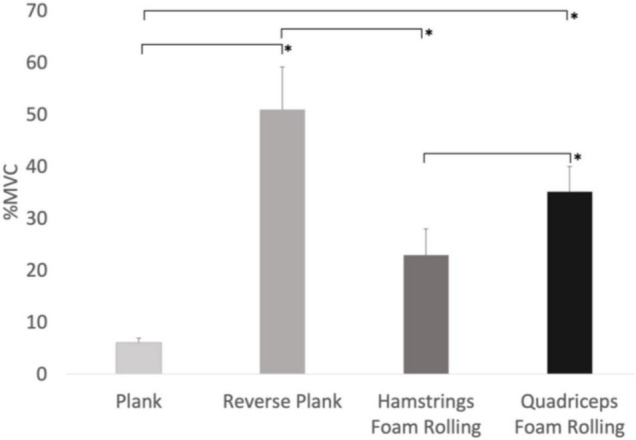
Main effect for conditions with the upper lumbar erector spinae EMG activity. *Significant difference between the conditions (*p* < 0.05), %MVIC refers to MVICmax.

A main effect for condition was observed for ULES muscle activity (*F*_3,24_ = 19.801, p < 0.001, Pη2 = 0.712) with reverse plank (*p* = 0.003, *d* = 12.06), and quadriceps foam rolling (*p* = 0.002, *d* = 7.84) exhibiting 746%, and 482% greater ULES activity compared to the prone plank. Moreover, ULES muscle activity during reverse plank (*p* = 0.004, *d* = 7.64), and quadriceps foam rolling (*p* = 0.020, *d* = 2.25) was 122% and 53% higher compared to hamstrings foam rolling, respectively (Figure 5). No main effect for time (*F*_2,16_ = 1.252, *p* = 0.312) nor interaction effect for time × condition (*F*_6,48_ = 0.840, *p* = 0.454) was observed for ULES muscle activity.

### Lumbo-Sacral Erector Spinae Muscles

**FIGURE 6:**
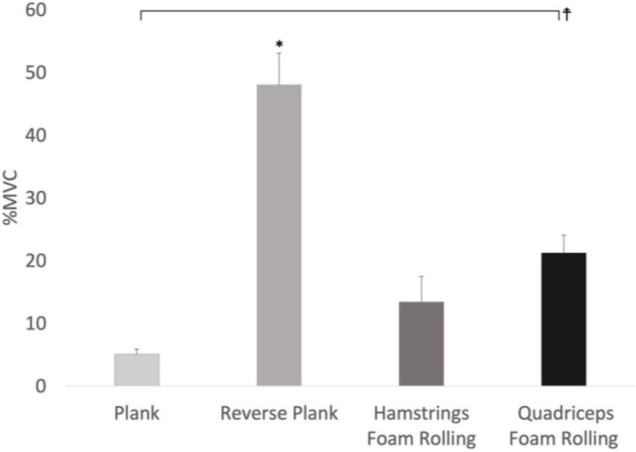
Main effect for conditions with lumbo-sacral erector spinae muscle EMG activity. *Significance difference between reverse plank with plank, hamstrings foam rolling, and quadriceps foam rolling (*p* < 0.05). ^?^Significant difference between the conditions (*p* < 0.05), %MVIC refers to MVICmax.

A main effect for condition was observed for LSES muscle activity (*F*_3,24_ = 34.522, p < 0.001, Pη2 = 0.812) with reverse plank exhibiting 855%, 259% and 126% greater LES activity compared to the prone plank (p < 0.001, *d* = 7.68), hamstrings foam rolling (*p* = 0.002, *d* = 4.11), and quadriceps foam rolling (*p* = 0.005, *d* = 2.34), respectively (Figure 6). Moreover, LSES muscle activity during quadriceps foam rolling was 321% higher compared to prone plank (*p* = 0.003, 8.27). No main effect for time (*F*_2,16_ = 2.758, *p* = 0.075) nor interaction effect for time × condition (*F*_6,48_ = 2.937, *p* = 0.368) was observed for LSES muscle activity.

## Discussion

The major findings of the study were that higher ULES muscle activation can be achieved with reverse planks and quadriceps foam rolling than with prone planks or hamstrings foam rolling. Secondly, LSES EMG activity was also higher with reverse planks significantly exceeding prone planks, quadriceps and hamstrings foam rolling. lower abdominal muscles experienced greater activation with quadriceps foam rolling vs. reverse plank or hamstrings foam rolling. The prone plank exhibited greater muscle activation only with the external obliques when compared to quadriceps or hamstrings foam rolling or reverse planks. Hence, although prone planks are a ubiquitously popular exercise for training the core muscles, reverse planks seem to provide greater activation of back muscles. However, prone plank exercises are more effective at activating the external obliques but are not significantly different than quadriceps foam rolling for lower abdominals activation.

From a performance perspective, vertebral column stability and the effective transfer of torques and angular momentum through the kinetic chain are dependent upon a strong core or trunk musculature (Kibler et al., 2006; Behm et al., 2010a; Behm and Colado Sanchez, 2013). From a health perspective, adequate levels of core muscle activation are necessary to attenuate risk of injury and muscle soreness (Willardson, 2007a,b; Behm et al., 2010a,^2011^, ^2015^). Low back pain is one of the leading musculoskeletal health problems internationally (Steffens et al., 2016) and is associated with lumbar extensors deconditioning (Behennah et al., 2018). Hence, numerous exercises and training techniques for the trunk muscles are commonly prescribed to specifically activate the core in order to achieve greater strength and endurance (Parfrey et al., 2008, 2014; van den Tillaar and Saeterbakken, 2018).

Although, the prone plank is a popular exercise to train the core, the dorsal core muscles monitored in this study (ULES and LSES) had EMG values that averaged less than 10% of the activation associated with a MVIC exercise [maximum isometric back extension from a prone position on a table similar to a Biering Sorensen test (Pitcher et al., 2007, 2008)] for these muscles (Figure 4). The prone plank position was held for 30-s by recreationally trained individuals and it is likely that if held till task failure, or performed by untrained participants, back muscle activation would have increased. However, over the 30-s period there were no significant main effects for time or time x condition interactions and thus reverse planks provided greater dorsal core EMG activity (∼50% of MVIC).

In contrast, the prone plank did provide higher muscle activation for the external obliques (∼40%) vs. the reverse plank (∼5% of MVIC) and foam rolling (∼10–15% of MVIC) (Figure 2). When fatigue is experienced during a prone plank, it is common to see a bow in the trunk (increasing trunk concavity) that results in a lowering of the pelvis toward the floor. While the external obliques function to rotate and laterally flex the trunk, they also contribute to spinal stability (Basmaijan, 1981). While quadriceps foam rolling provided the greatest lower abdominals EMG activity (∼50% of MVIC), there was no significant difference compared to the plank. Although the prone plank is generally considered a training exercise for the core (both ventral and dorsal trunk), the present results suggest that it is more effective for activating the ventral core muscles (external obliques and lower abdominals).

It is quite common for individuals when training to add additional core exercises (Willardson, 2007b; Hibbs et al., 2008). However, it is consistently documented that a lack of time is perceived as a significant barrier to regular exercise (Canadian Society for Exercise Physiology, 2003) and thus exercises that integrate core muscle groups with other muscle strengthening exercises could decrease exercise time. Whilst, incorporating quadriceps foam rolling into an exercise program can increase hip and knee ROM (MacDonald et al., 2013; Behm and Wilke, 2019; Behm et al., 2020; Wilke et al., 2020), the present results demonstrate that it is also as effective or more effective than a core specific exercise such as prone planks for activating core musculature such as the ULES, LSES, and lower abdominals.

These findings are in accord with other studies that have also demonstrated high core muscle activation with metastable, closed kinetic chain exercises (Anderson and Behm, 2005a,b; Behm et al., 2010a,^2005^), whose primary function may not be considered to be core strengthening. For example, Behm et al. (2009) had participants run on a treadmill for 30 min at 60 or 80% of their maximum heart rate reserve. They reported that the running elicited greater ULES and LSES EMG activity than a Biering-Sorensen back extension exercise. Running can be considered a metastable activity (Kibele et al., 2015) (sequential transitions from a stable landing to relatively unstable flight phase), with core activation essential to stabilize the hips and vertebral column during unilateral landing and propulsions as well as the flight phases (Behm et al., 2009). Hence, running can be employed as an efficient, multifunctional exercise combining cardiovascular and trunk endurance benefits without the need for additional trunk strengthening exercises. Another example is provided by the results of Hamlyn et al. (2007), who reported greater activity of the LSES and ULES during 80% one repetition maximum squats and deadlifts, which exceeded the level of muscle activity of body weight squats or a selection of exercises performed on an unstable exercise ball (superman and side bridge). These findings are similar to van den Tillaar and Saeterbakken (2018) who reported non-significant muscle activation differences between 6-repetition maximum back squats and a prone bridge exercise with the rectus abdominis or external oblique, however, higher erector spinae activation with squatting. Whilst foam rolling may not be considered as highly unstable, it does involve sequentially balancing and moving the center of gravity over and outside the foam roller base of support. The trunk must maintain some degree of metastability to efficiently perform this exercise.

Hamstrings foam rolling was least effective overall, eliciting approximately 10–20% of MVIC EMG activity for the four tested muscles. While the planks and quadriceps foam rolling necessitated generally higher core activation to maintain an extended trunk position, hamstrings foam rolling was performed in a flexed hip position. This relatively seated position would not have necessitated high EMG activity to maintain an erect trunk position.

## Conclusion

The traditional prone plank exercise induced less muscle activation of the dorsal core muscles (ULES and LSES) than reverse planks or quadriceps foam rolling. Prone plank exercises were effective at activating the ventral core muscles (external obliques and lower abdominals). Hamstrings foam rolling was generally the least effective exercise for activating the core muscles. Therefore, while reverse plank exercises are quite effective at activating dorsal core muscles, individuals who perform quadriceps foam rolling and wish to be time efficient may still induce back muscle training stress without additional core exercises such as planks.

## Data Availability Statement

The raw data supporting the conclusions of this article will be made available by the authors, without undue reservation.

## Ethics Statement

The studies involving human participants were reviewed and approved by Memorial University of Newfoundland Interdisciplinary Committee on Ethics in Human Research (ICEHR). The patients/participants provided their written informed consent to participate in this study. Written informed consent was obtained from the individual(s) for the publication of any identifiable images or data included in this article.

## Author Contributions

All authors listed have made a substantial, direct, and intellectual contribution to the work, and approved it for publication.

## Conflict of Interest

The authors declare that the research was conducted in the absence of any commercial or financial relationships that could be construed as a potential conflict of interest.

## Publisher’s Note

All claims expressed in this article are solely those of the authors and do not necessarily represent those of their affiliated organizations, or those of the publisher, the editors and the reviewers. Any product that may be evaluated in this article, or claim that may be made by its manufacturer, is not guaranteed or endorsed by the publisher.

## Abbreviations

ANOVA: analysis of variance
EMG: electromyography
LSES: lumbosacral erector spinae
MVIC: maximum voluntary isometric contraction
RMS: root mean square
ROM: range of motion
ULES: upper lumbar erector spinae.
